# LLPS-Driven Membraneless Organelle Activity Landscape Reveals Molecular Subtypes, Immune States, and Prognostic Stratification in Lung Adenocarcinoma

**DOI:** 10.3390/ijms272110008

**Published:** 2026-11-08

**Authors:** Hongmei Yan, Nanyan Yang, Lu Zhang, Haorui Tian, Wei Mo, Maoying Lan, Zilong Lin, Hantan Lin, Zeyu Wang, Juntao Zou, Fen He, Jiancong Sun

**Affiliations:** 1 Department of Radiation Oncology, The First Affiliated Hospital of Guangzhou Medical University, Guangzhou 510000, China; yanhongmei126@i.smu.edu.cn (H.Y.); zhanglu_gyfyy@163.com (L.Z.); hrtian2000@163.com (H.T.); weimo081380@163.com (W.M.); zhenhualmy@163.com (M.L.); linzlemail@163.com (Z.L.); linhantan2021@163.com (H.L.); zeeeyu@126.com (Z.W.); 2 Department of Thoracic Surgery and Oncology, The First Affiliated Hospital of Guangzhou Medical University, China State Key Laboratory of Respiratory Disease & National Clinical Research Center for Respiratory Disease, Guangzhou Institute of Respiratory Disease, Guangzhou 510165, China; annieyang1994@163.com; 3 Zhongshan School of Medicine, Sun Yat-Sen University, Guangzhou 510080, China; zoujuntao@163.com

**Keywords:** lung adenocarcinoma, phase separation, molecular subtypes, immunity, prognostic stratification

## Abstract

Liquid–liquid phase separation (LLPS) drives the formation of membraneless organelles (MLOs) that regulate cellular compartmentalization and diverse biological processes. However, the landscape and clinical relevance of MLO-associated activity in lung adenocarcinoma (LUAD) remain unclear. We integrated curated MLO-associated gene sets with transcriptomic data to estimate MLO activity using single-sample gene set enrichment analysis. MLO-related molecular subtypes were identified via consensus clustering, and an integrative machine learning framework was used to develop and validate the Membraneless Organelle Risk Score (MLORS) across independent cohorts. We further examined associations of MLORS with immune features, genomic alterations, and predicted treatment sensitivity and evaluated the function and phase-separation properties of E2F7 in vitro. MLO-associated activity was broadly dysregulated in LUAD, with high-MLO and low-MLO subtypes exhibiting distinct immune profiles and survival outcomes. High MLORS was associated with poorer survival, a computationally inferred immune-suppressed microenvironmental profile, higher tumor mutational burden, and lower predicted sensitivity to selected EGFR-targeted agents and chemotherapy. E2F7 formed nuclear condensates with LLPS-like properties and promoted lung cancer cell proliferation and migration in vitro. These findings link transcriptionally inferred MLO activity to the heterogeneity and prognosis of LUAD, support MLORS as a candidate prognostic stratification tool, and identify E2F7 phase-separation behavior as a potential therapeutic vulnerability.

## 1. Introduction

Lung adenocarcinoma (LUAD)—the most common histological subtype of non-small cell lung cancer [1]—is characterized by molecular and clinical heterogeneity, which underlies the marked variability in patient prognosis and therapeutic response [2]. Although advances in targeted therapies and immune checkpoint blockade have substantially improved outcomes in selected patient populations [3,4], the durable clinical benefit for these patients remains limited [5,6,7]. Consequently, accurate risk stratification and personalized therapeutic decision-making remain major clinical challenges, highlighting the need for integrative biomarkers that capture tumor biology beyond individual genomic alterations.

Liquid–liquid phase separation (LLPS) has emerged as a fundamental biophysical mechanism governing intracellular organization [8]. Through LLPS, cells assemble dynamic biomolecular condensates known as membraneless organelles (MLOs) [9], including nucleoli [10], stress granules [11], and nuclear speckles [12]. Rather than serving as passive compartments, these condensates dynamically concentrate or exclude specific macromolecules [13], thereby coordinating diverse biological processes including transcriptional regulation [14], RNA metabolism [15], signal transduction [16], and cellular stress responses [17]. Their highly dynamic and reversible nature enables cells to rapidly reorganize molecular interactions in response to developmental, metabolic, and environmental cues [18].

Increasing evidence suggests that dysregulated LLPS contributes to tumor initiation and progression by reshaping regulatory networks, sustaining oncogenic signaling, and promoting cellular plasticity [18,19]. Several oncogenic regulators, including YAP/TAZ, BRD4, and mutant p53, undergo phase separation to form biomolecular condensates that amplify transcriptional programs or disrupt normal regulatory control [20,21,22]. In addition, aberrant stress granule dynamics and RNA-binding protein-associated condensates have been implicated in therapeutic resistance, immune evasion, and tumor adaptation [23,24]. Nevertheless, existing studies have largely focused on individual phase-separating proteins or isolated condensates rather than the coordinated activity of MLOs at the systems level [25].

Consequently, the global organization and biological significance of MLO activity in LUAD remain poorly defined. Whether coordinated MLO activity delineates biologically and clinically meaningful tumor subtypes and how these activity states relate to tumor immunity, genomic alterations, and therapeutic response remain largely unknown. Given the compositional complexity and dynamic behavior of biomolecular condensates [13], gene set-based activity profiling provides a practical systems-level strategy for capturing coordinated MLO activity.

In this study, we systematically characterized LLPS-driven MLO activity in large LUAD transcriptomic cohorts by integrating 24 curated MLO-associated gene sets with single-sample gene set enrichment analysis (ssGSEA). We further developed and validated the Membraneless Organelle Risk Score (MLORS) using an integrative machine learning framework and investigated the mechanistic role of the representative regulator E2F7 in LLPS-mediated tumor progression. Through integrating systems-level activity profiling with mechanistic validation, this study establishes a framework for understanding the biological and clinical significance of MLO activity in LUAD and provides new insights into the roles of LLPS in tumor heterogeneity and precision oncology.

## 2. Results

### 2.1. Global Patterns of LLPS-Driven MLO Activity in LUAD

To examine the global activity landscape of liquid–liquid phase separation (LLPS)-driven membraneless organelles (MLOs) in lung adenocarcinoma (LUAD), we assembled 24 experimentally validated MLO gene sets and quantified their activity in the TCGA-LUAD cohort using single-sample gene set enrichment analysis (ssGSEA) (Figure S1A) [26].

**Figure 1:**
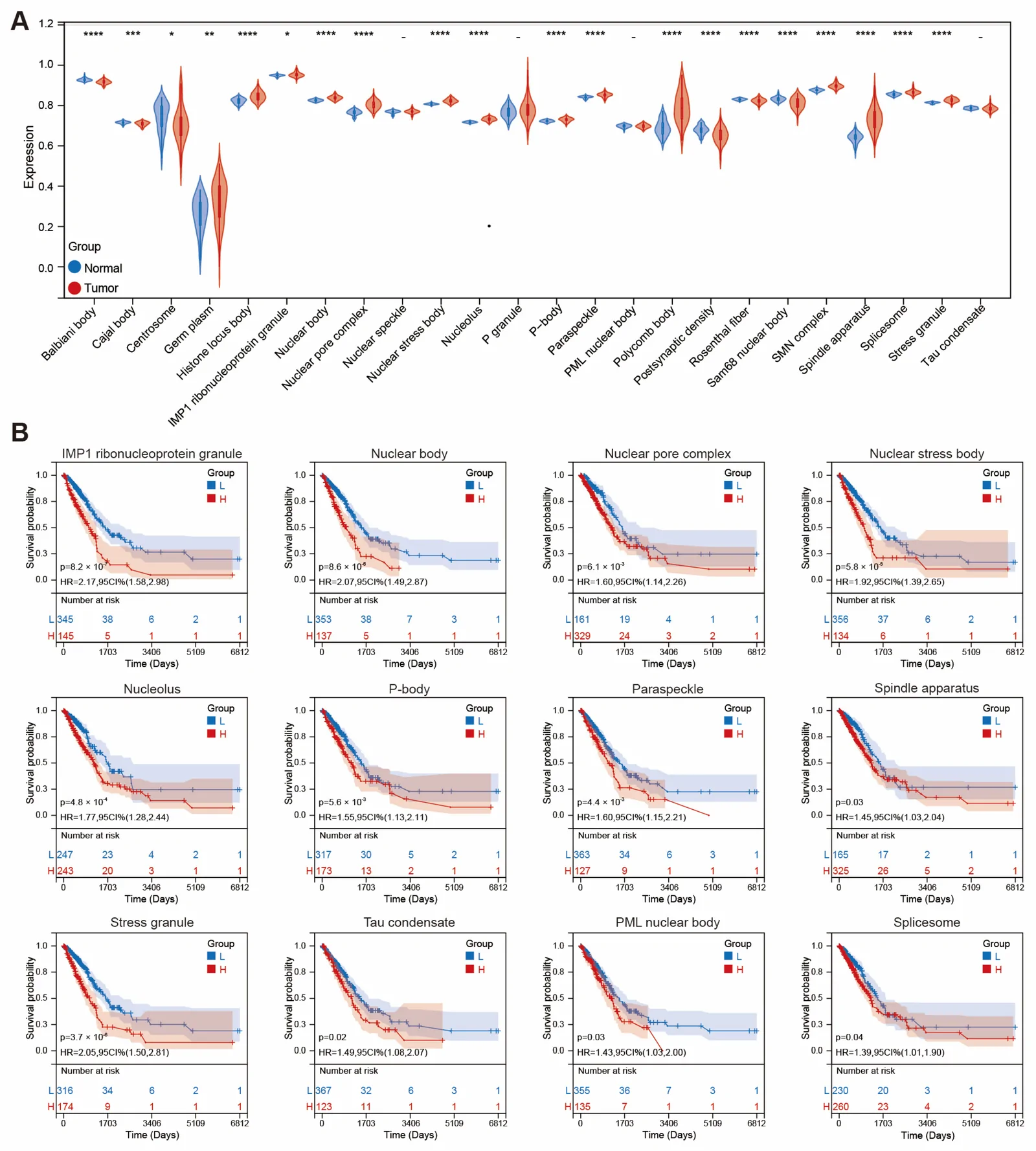
Global landscape and prognostic relevance of MLO-associated activity in LUAD. (**A**) Comparison of ssGSEA-derived activity scores for 24 MLO-associated gene sets between tumor tissues and adjacent normal tissues in the TCGA-LUAD cohort. (**B**) Kaplan–Meier survival curves showing overall survival according to the activity levels of 12 prognosis-associated MLOs. * represents *p* values < 0.05; ** represents *p* values < 0.01; *** represents *p* values < 0.001; and **** represents *p* values < 0.0001.

The comparison between tumor and adjacent normal tissues revealed widespread alterations in MLO activity across the LUAD samples. Twenty of the 24 MLOs exhibited significant activity differences, with 14 showing increased activity in tumors (Figure 1A), suggesting the pervasive dysregulation of LLPS-associated condensates in LUAD.

Next, we evaluated the association between MLO activity and patient survival. Twelve MLOs were significantly associated with overall survival (OS) (Figure 1B). After adjustment for covariates, multivariable Cox regression analysis identified spindle, IMP1 ribonucleoprotein granule, and stress granule activity as independent prognostic factors linked to poorer survival in LUAD patients (Figure S1B,C).

Taken together, these findings indicate that LLPS-driven MLO activity is broadly reprogrammed in LUAD and that specific condensates exhibit independent prognostic relevance.

### 2.2. Prognosis-Associated MLOs Exhibit Coordinated Activity Patterns in LUAD

**Figure 2:**
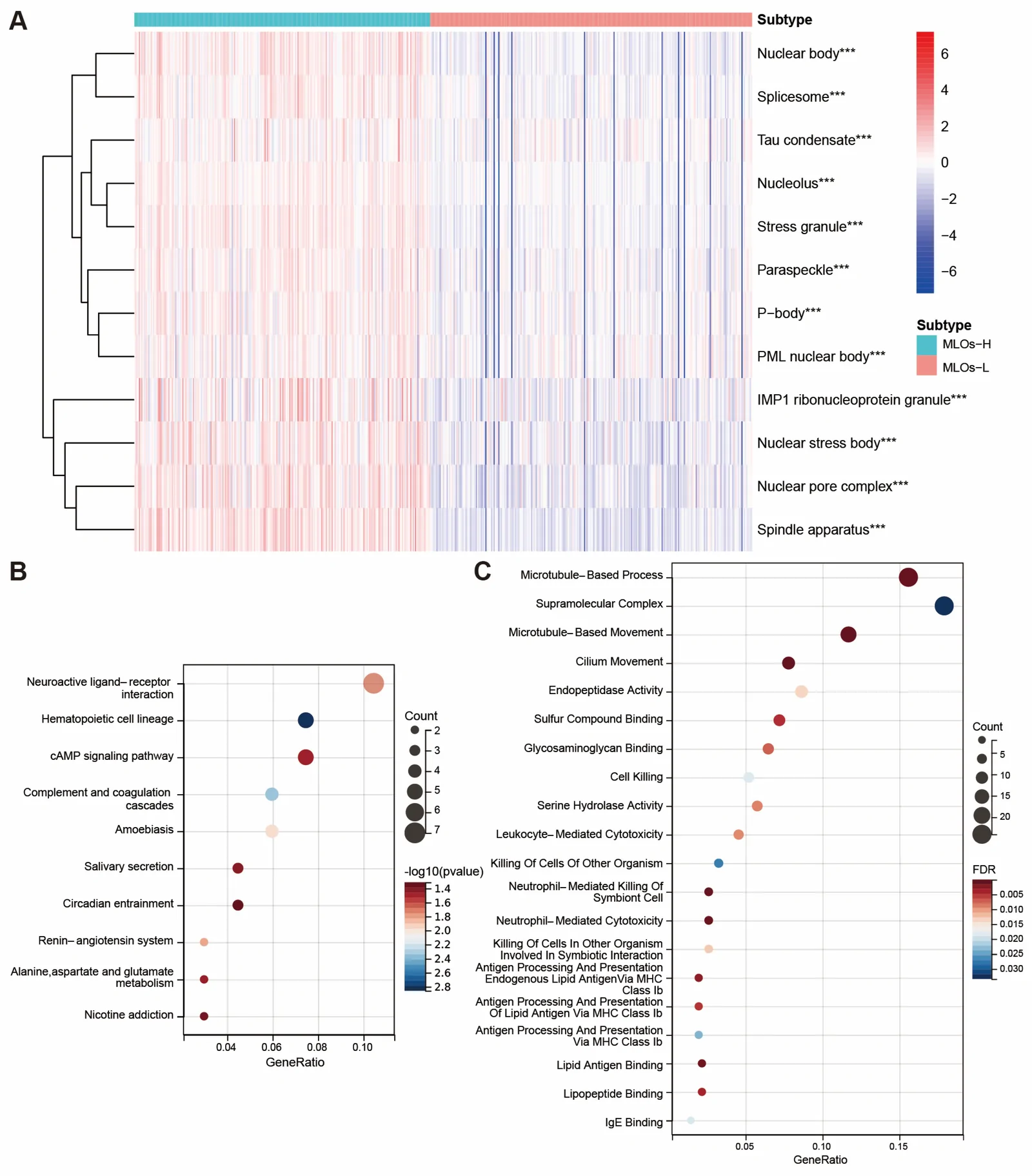
Coordinated transcriptional patterns of prognosis-associated MLO gene sets and their functional characteristics. (**A**) Heatmap showing the activity patterns of the 12 prognosis-associated MLOs between MLOs-H and MLOs-L subtypes. (**B**) KEGG pathway enrichment analysis of genes differentially expressed between the two subtypes. (**C**) GO biological process enrichment analysis of subtype-associated genes. *** represents *p* values < 0.001.

To characterize whether coordinated transcriptional patterns among prognosis-associated MLO gene sets were present in LUAD, we performed consensus clustering based on the ssGSEA-derived profiles of the 12 prognosis-associated MLOs in the TCGA-LUAD cohort (*n* = 490). The consensus cumulative distribution function (CDF) and delta-area analyses supported an optimal two-cluster solution (k = 2), defining two distinct MLO activity subtypes (Figure S1D). One subtype exhibited consistently higher ssGSEA-inferred MLO-associated transcriptional scores across the 12 prognosis-associated MLO gene sets and was designated MLOs-high (MLOs-H), whereas the other displayed lower scores and was designated MLOs-low (MLOs-L). All 12 MLOs significantly differed in activity between the two subtypes (Figure 2A).

Functional enrichment analysis revealed marked biological differences between the two subtypes. KEGG analysis identified a significant enrichment of signaling, metabolic, and immune-related pathways (Figure 2B). Consistently, Gene Ontology analysis demonstrated the differential enrichment of cytoskeleton-related processes, including microtubule-dependent movement and cilium organization, together with immune-associated biological processes such as antigen processing and presentation and cytotoxicity (Figure 2C).

These findings indicate that MLO activity-based stratification captures biologically distinct LUAD subtypes characterized by divergent functional programs and immune states.

### 2.3. Immune Microenvironment Differences Associated with MLO Activity Subtypes

**Figure 3:**
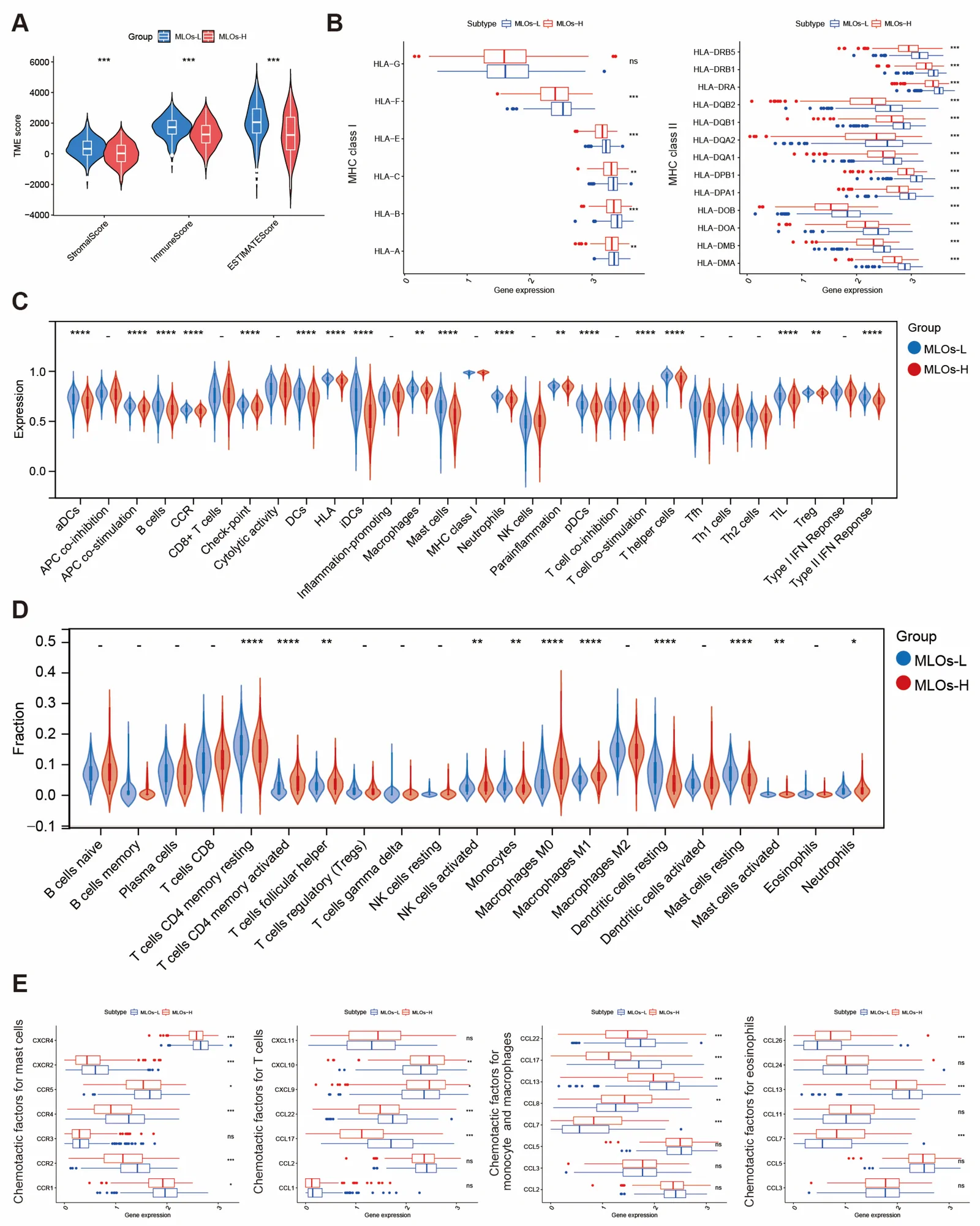
Immune microenvironment characteristics of MLO-associated subtypes. (**A**) Comparison of ESTIMATEScore, ImmuneScore, and StromalScore between MLOs-H and MLOs-L subtypes. (**B**) Expression of major histocompatibility complex (MHC) class I and class II genes in the two subtypes. (**C**) Comparison of ssGSEA-derived scores for 29 immune-related pathways between MLOs-H and MLOs-L tumors. (**D**) Relative proportions of 22 immune-cell populations inferred using the CIBERSORT algorithm in MLOs-H and MLOs-L tumors. (**E**) Comparison of selected chemokines involved in immune-cell recruitment, including chemokines associated with mast cells, T cells, monocytes/macrophages, and eosinophils, between the two subtypes. * represents *p* values < 0.05; ** represents *p* values < 0.01; *** represents *p* values < 0.001; and **** represents *p* values < 0.0001.

To delineate the immune microenvironment associated with MLO activity-defined subtypes, the ESTIMATE algorithm was applied. Relative to the MLOs-L subtype, tumors classified as MLOs-H exhibited significantly lower ESTIMATEScore, ImmuneScore and StromalScore values, indicating lower computationally inferred immune and stromal content in bulk transcriptomic profiles (Figure 3A).

Analysis of immune checkpoint molecules revealed that both inhibitory receptors and co-stimulatory molecules were expressed at higher levels in the MLOs-L subtype (Figure S2A,B). Likewise, the expression of major histocompatibility complex (MHC) class I and class II genes was also consistently elevated in the MLOs-L tumors, suggesting enhanced antigen presentation activity (Figure 3B). In agreement with these findings, ssGSEA of 29 immune-related pathways demonstrated significantly higher activity of 16 pathways in the MLOs-L subtype, including cytokine-cytokine receptor interaction, T cell receptor signaling, and interferon-γ response (Figure 3C) [27]. Together, these findings are consistent with a more immune-active transcriptional profile in the MLOs-L subtype.

CIBERSORT-based computational deconvolution of bulk transcriptomic profiles further indicated differences in the estimated relative abundance of immune-cell populations between the subtypes. The MLOs-L subtype showed higher estimated relative proportions of resting mast cells, monocytes/macrophages, resting dendritic cells, and resting CD4+ memory T cells, whereas the MLOs-H subtype showed higher proportions of activated CD4+ memory T cells, follicular helper T cells, activated natural killer cells, M0 and M1 macrophages (Figure 3D). The analysis of chemokine expression supported these findings, revealing increased levels of mast cell-associated chemokines in the MLOs-L subtype (Figure 3E).

The assessment of immunogenomic features demonstrated that the tumor mutational burden (TMB) was significantly higher in the MLOs-H subtype compared with the MLOs-L subtype, while the microsatellite instability (MSI) status did not differ significantly between groups (Figure S2C,D).

Taken together, these results indicate that the transcriptionally defined MLO-associated groups are associated with distinct computationally inferred immune-cell composition scores, immune pathway signatures, and immunogenomic features, underscoring the close relationship between MLO activity states and the immune contexture of LUAD.

### 2.4. Genomic Alteration Profiles and Predicted Drug Sensitivity Associated with MLO Activity Subtypes

To determine whether distinct MLO activity states are accompanied by differences in genomic architecture and therapeutic vulnerability, we compared somatic mutational landscapes between the MLOs-H and MLOs-L subtypes (Figure S3A). Across both groups, missense mutations and single-nucleotide variants represented the dominant alteration types; however, the overall mutation burden was significantly higher in the MLOs-H subtype.

**Figure 4:**
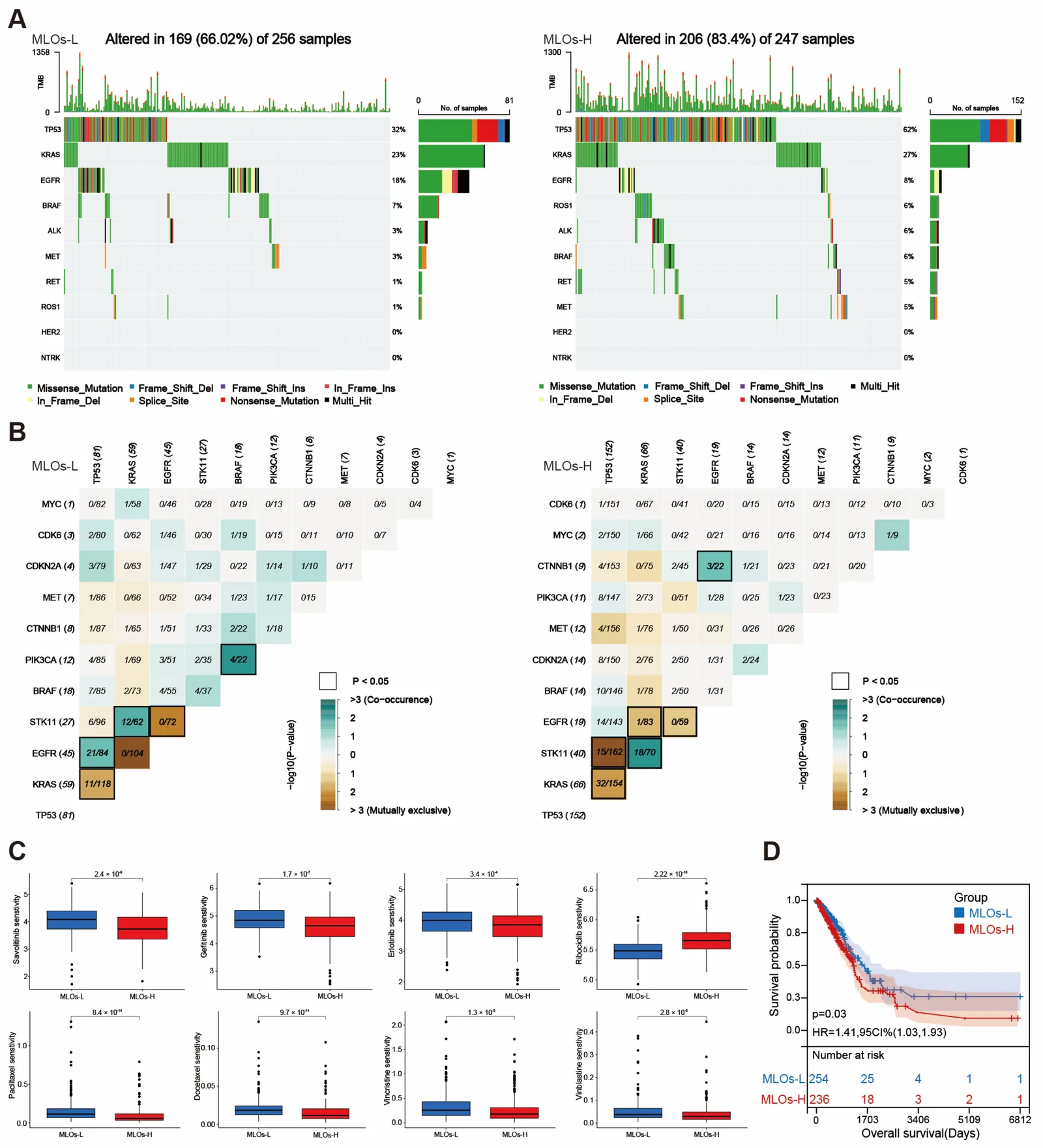
Genomic alterations, predicted drug sensitivity, and survival outcomes of MLO-associated subtypes. (**A**) Comparison of mutation frequencies of ten clinically relevant LUAD driver genes between MLOs-H and MLOs-L tumors. (**B**) Comparison of recurrent co-mutation patterns between the two subtypes. (**C**) Predicted sensitivity to representative targeted therapies and chemotherapeutic agents estimated using the oncoPredict algorithm. (**D**) Kaplan–Meier survival curves of overall survival in MLOs-H and MLOs-L tumors.

Evaluation of ten clinically relevant driver genes in lung adenocarcinoma (*EGFR*, *ALK*, *KRAS*, *ROS1*, *BRAF*, *MET*, *RET*, *HER2*, *NTRK*, and *TP53*) revealed marked subtype-specific mutation patterns [28]. The mutation frequencies of nine genes, including *TP53*, *KRAS*, and *BRAF*, were significantly elevated in the MLOs-H subtype, whereas *EGFR* mutations were preferentially observed in MLOs-L tumors (Figure 4A).

Furthermore, the co-mutation analysis highlighted distinct combinatorial architectures, with the *EGFR-TP53* co-alteration pattern occurring more frequently in the MLOs-L subtype, while co-mutation signatures associated with aggressive tumor phenotypes, such as *KRAS-LKB1*, *KRAS-TP53*, and *TP53-LKB1*, were significantly overrepresented in the MLOs-H subtype (Figure 4B) [29,30,31].

To investigate the therapeutic implications of these genomic differences, computational drug sensitivity prediction was performed using the oncoPredict algorithm. This analysis uncovered divergent patterns of predicted therapeutic susceptibility across MLO activity subtypes. Specifically, the MLOs-L subtype exhibited higher predicted sensitivity to the EGFR-targeted agents gefitinib and erlotinib, consistent with its enrichment for *EGFR* mutations, and to the MET inhibitor savolitinib [32] (Figure 4C). In contrast, the MLOs-H subtype displayed increased predicted susceptibility to the cyclin-dependent kinase 4/6 (CDK4/6) inhibitor ribociclib [33], a pattern that may reflect underlying cell-cycle dysregulation associated with frequent *TP53* alterations (Figure 4C) [34]. Beyond targeted therapies, the MLOs-L subtype also demonstrated a comparatively higher predicted sensitivity to several conventional cytotoxic chemotherapeutic agents, including paclitaxel, docetaxel, vincristine, and vinblastine (Figure 4C) [35]. Consistent with these molecular and predicted therapeutic distinctions, the Kaplan–Meier survival analysis revealed a significantly improved overall survival among patients classified as MLOs-L compared with those in the MLOs-H subtype, underscoring the clinical relevance of stratification based on MLO activity (Figure 4D).

### 2.5. Construction and Validation of an Integrated Multi-Algorithm Prognostic Risk Score

**Figure 5:**
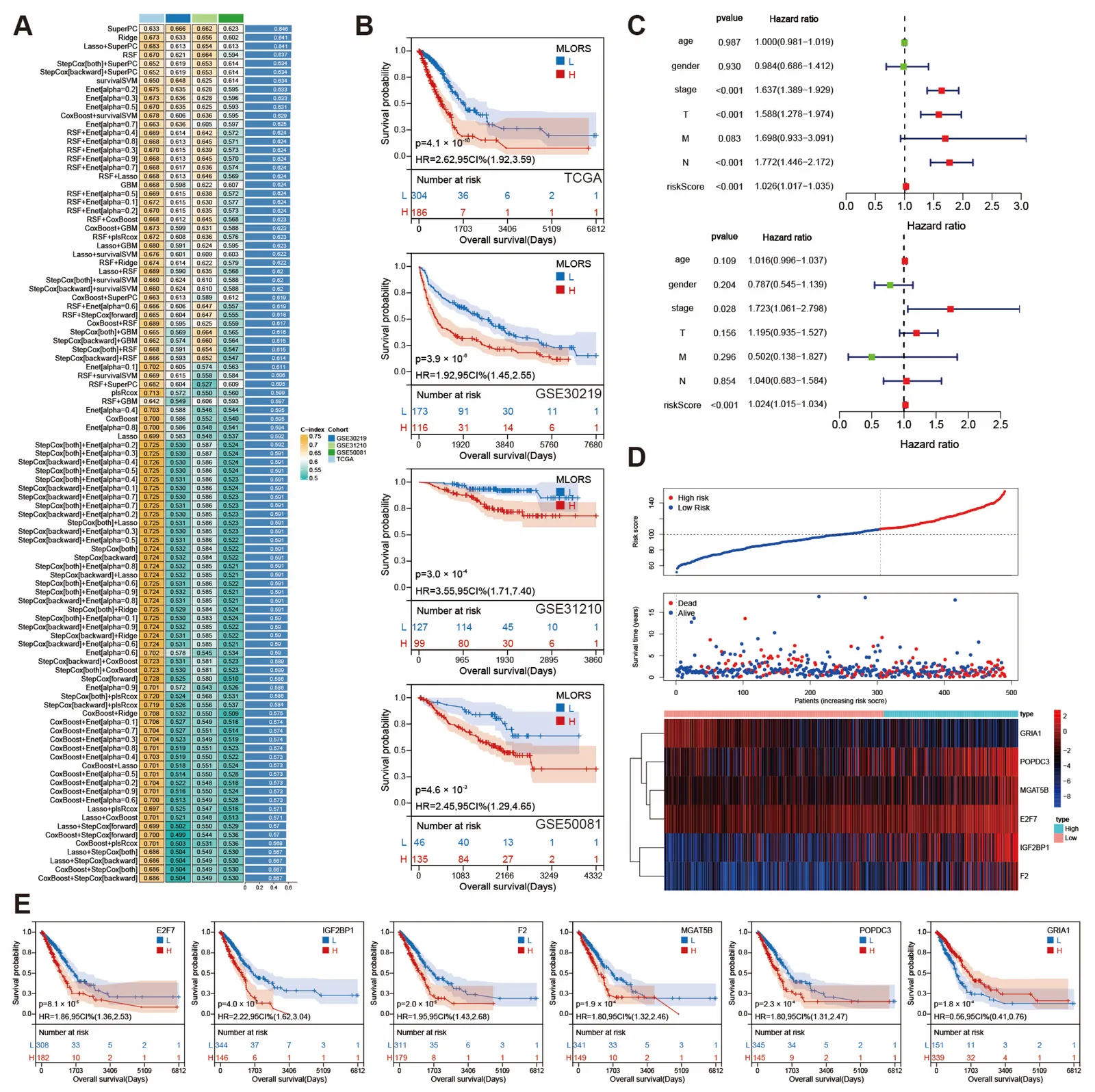
Construction, validation, and prognostic performance of MLO-based risk score (MLORS). (**A**) Performance comparison of survival models generated using ten machine-learning algorithms under 10-fold cross-validation, with the Random Survival Forest (RSF) model selected as the final model. (**B**) Kaplan–Meier survival curves comparing overall survival between MLORS high- and low-risk groups in the training and validation cohorts. (**C**) Univariate (upper) and multivariable Cox (lower) regression analyses evaluating the independent prognostic contribution of MLORS relative to clinicopathological variables. (**D**) Distribution of MLORS values, survival status, and risk-group classification in the training cohort. (**E**) Kaplan–Meier curves showing the prognostic associations of the six genes included in the MLORS model.

To derive a prognostic model reflecting MLO activity, univariate Cox regression analysis was conducted on genes differentially expressed between the MLOs-H and MLOs-L subtypes within the TCGA-LUAD cohort, identifying 117 prognostic genes significantly associated with overall survival (OS) (Figure S3B). The TCGA-LUAD cohort served as the training cohort, whereas the independent datasets GSE30219, GSE31210, and GSE50081 served as external validation cohorts. An integrated machine learning framework comprising 10 survival modeling algorithms was applied, generating 101 candidate models that were evaluated using 10-fold cross-validation. Comparative performance analysis showed that all candidate models achieved C-index values above 0.5 in the TCGA training cohort (Figure 5A). Considering both gene number and C-index, RSF was selected as the optimal model because it combined a high C-index (0.670) with the fewest genes (six), model simplicity, and a low overfitting risk (Figure 5A and Table S1). The locked model was then applied unchanged to GSE30219, GSE31210, and GSE50081, yielding C-index values of 0.621, 0.664, and 0.594, respectively, supporting its generalizability (Figure 5A).

Based on this optimal RSF model, a composite prognostic signature—termed the Membraneless Organelle Risk Score (MLORS)—was established using six genes (*MGAT5B*, *E2F7*, *IGF2BP1*, *F2*, *POPDC3*, and *GRIA1*). MLORS consistently stratified patients into distinct risk groups across all cohorts. Kaplan–Meier survival analysis demonstrated that patients in the high-risk group exhibited significantly poorer overall survival than those in the low-risk group (Figure 5B). Time-dependent receiver operating characteristic (ROC) analysis showed that MLORS achieved AUC values of 0.72, 0.70, and 0.71 for predicting 1-, 3-, and 5-year overall survival, respectively (Figure S4A). Notably, its predictive performance at 3 years exceeded that of conventional clinicopathological variables, including age, sex, and TNM stage (Figure S4B). Multivariable Cox regression analysis further confirmed MLORS as an independent prognostic factor for OS in patients with LUAD (Figure 5C).

Analysis of the MLORS distribution revealed that higher risk scores were accompanied by an increased number of death events (Figure 5D). At the gene level, elevated expression of *MGAT5B*, *E2F7*, *IGF2BP1*, and *F2* was associated with higher MLORS values and poorer survival, whereas *POPDC3* and *GRIA1* exhibited the opposite trend (Figure 5D,E). In particular, higher *GRIA1* expression was associated with prolonged survival (Figure 5E).

Together, these results indicate that MLORS provides a robust and clinically meaningful prognostic model that captures MLO activity-related heterogeneity beyond conventional clinicopathological parameters.

### 2.6. Association of MLORS with an Immunosuppressive Microenvironment and Elevated Tumor Mutational Burden

**Figure 6:**
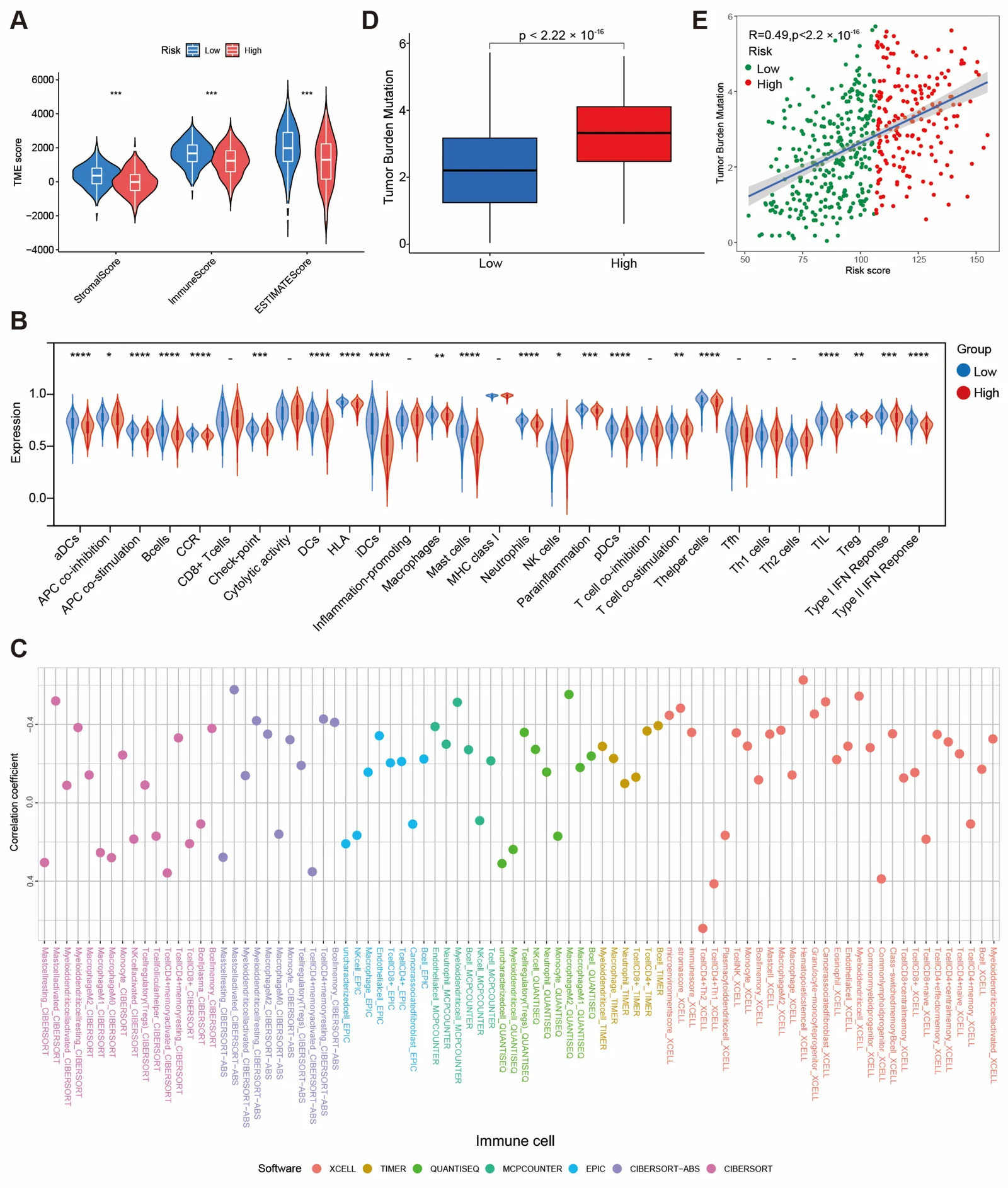
Immune microenvironment and immunogenomic features associated with MLORS. (**A**) Comparison of ESTIMATEScore, ImmuneScore, and StromalScore between MLORS high- and low-risk groups. (**B**) Comparison of ssGSEA-derived scores for 29 immune-related pathways between high- and low-risk groups. (**C**) Spearman correlation analysis between MLORS and immune-cell infiltration quantified using multiple deconvolution methods. (**D**) Comparison of TMB between MLORS risk groups. (**E**) Correlation between MLORS and TMB. * represents *p* values < 0.05; ** represents *p* values < 0.01; *** represents *p* values < 0.001; and **** represents *p* values < 0.0001.

To determine whether the prognostic differences captured by MLORS were accompanied by distinct immune microenvironment features, the quantitative evaluation of the tumor microenvironment revealed that the high-risk MLORS group exhibited significantly lower ImmuneScore, StromalScore, and ESTIMATEScore value than the low-risk group, consistent with an immune-desert-like computational profile resembling the MLOs-H subtype (Figure 6A).

The analysis of immune checkpoint molecules showed that most differentially expressed immune checkpoint genes were upregulated in the low-risk group (Figure S4C,D). Similarly, ssGSEA demonstrated significantly higher activity in 20 of the 29 immune-related pathways in the low-risk group (Figure 6B) [27]. Furthermore, integrative immune infiltration analyses demonstrated a significant negative correlation between MLORS and the abundance of the majority of immune cell populations (Figure 6C).

Collectively, these findings indicate that the low-risk group displays a more immune-active bulk-transcriptomic profile, whereas elevated MLORS is associated with lower computationally inferred immune-cell and stromal scores, consistent with an immune-suppressed profile, providing a mechanistic immunological context for its unfavorable prognostic association [36].

Consistent with these immune characteristics, the high-risk MLORS group exhibited a significantly higher tumor mutational burden (TMB) than the low-risk group, with MLORS showing a moderate positive correlation with TMB (R = 0.49, *p* < 0.001) (Figure 6D,E). In contrast, microsatellite instability (MSI) status did not differ significantly between the two risk groups (Figure S4E,F).

### 2.7. Genomic Alterations and Differential Therapeutic Sensitivity Associated with MLORS Risk Groups

**Figure 7:**
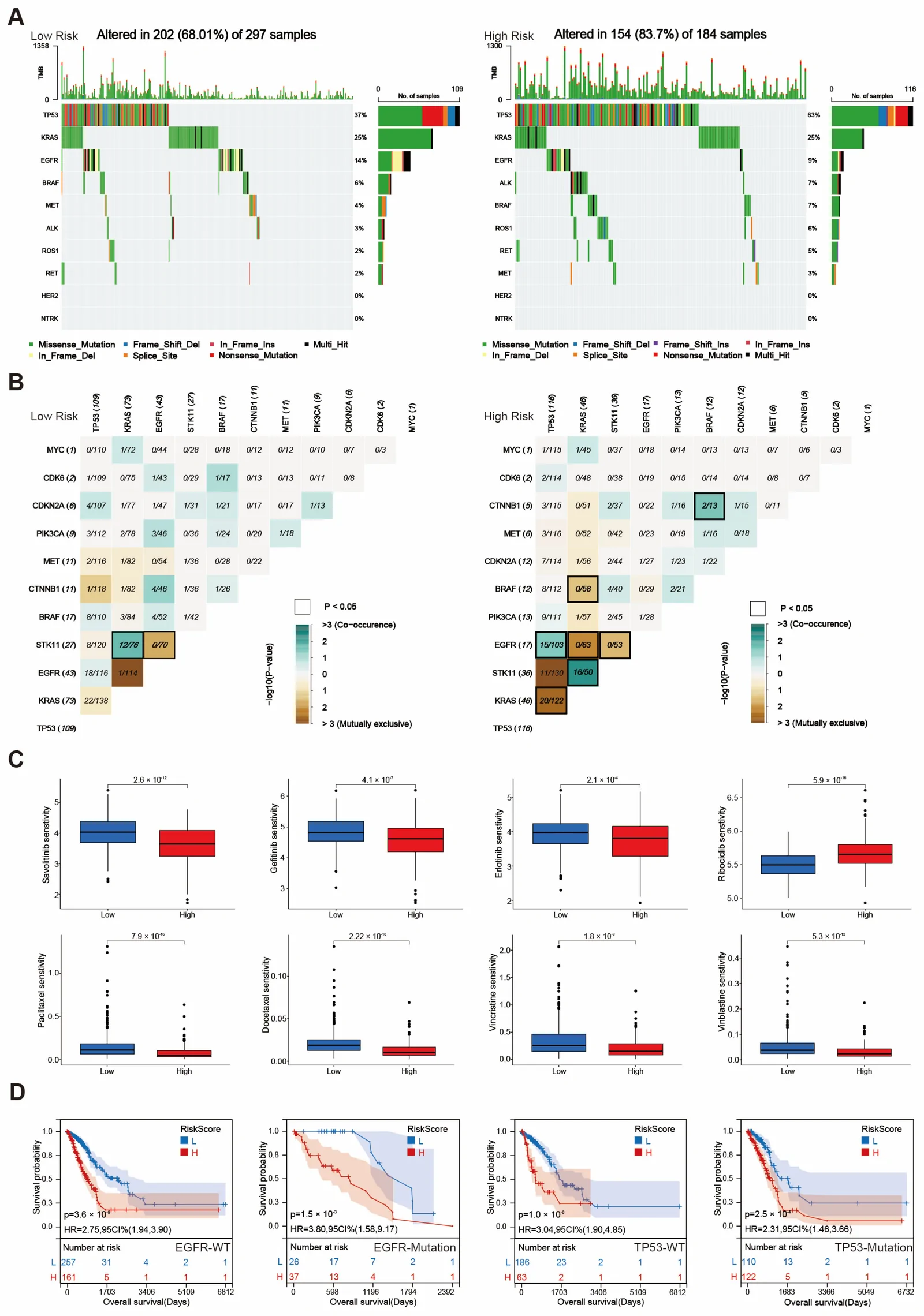
Genomic alterations and predicted therapeutic sensitivity associated with MLORS risk groups. (**A**) Comparison of mutation frequencies of key LUAD driver genes between MLORS high- and low-risk groups. (**B**) Comparison of recurrent co-mutation patterns associated with MLORS stratification. (**C**) Predicted sensitivity to representative targeted therapies and chemotherapeutic agents between MLORS risk groups. (**D**) Kaplan–Meier survival analysis of overall survival according to MLORS risk group in *EGFR*-mutant, *EGFR*-wild-type, *TP53*-mutant, and *TP53*-wild-type subgroups.

To further characterize the molecular basis underlying MLORS-defined risk stratification, the somatic mutation profiles revealed a significantly higher overall mutation burden in the high-risk MLORS group, despite similar distributions of mutation classes between the two groups (Figure S5A). Among clinically relevant driver genes, alterations in *TP53*, *ALK*, *BRAF*, *ROS1*, *RET*, and *MET* were enriched in the high-risk group, whereas *EGFR* mutations occurred more frequently in the low-risk group [28]. The *KRAS* mutation frequencies were comparable between the two groups (Figure 7A). Co-mutation analysis further demonstrated the enrichment of genomic configurations associated with aggressive tumor phenotypes, including *KRAS-LKB1*, *KRAS-TP53*, and *TP53-LKB1*, in the high-risk group, whereas *EGFR-TP53* co-mutation occurred slightly more frequently in the low-risk group (Figure 7B) [29,30,31].

Consistent with these genomic characteristics, drug response prediction revealed distinct therapeutic vulnerabilities between the two risk groups. The low-risk group was predicted to be more sensitive to EGFR-targeted therapies (gefitinib and erlotinib), the MET inhibitor savolitinib [32], and multiple chemotherapeutic agents [35], whereas the high-risk group exhibited greater predicted sensitivity to the CDK4/6 inhibitor ribociclib (Figure 7C) [33].

To determine whether MLORS provides prognostic value beyond established genomic biomarkers, patients were stratified by *EGFR* or *TP53* mutation status. High MLORS remained significantly associated with poorer overall survival in both the mutant and wild-type subgroups for *EGFR* and *TP53* (Figure 7D), demonstrating that the prognostic value of MLORS is independent of *EGFR* and *TP53* mutation status.

Collectively, MLORS identifies biologically distinct LUAD subsets with coordinated differences in genomic architecture, therapeutic vulnerability, and clinical prognosis, providing a framework for more refined risk stratification and personalized therapeutic decision-making.

### 2.8. E2F7 Forms Dynamic Condensates with LLPS-Consistent Biophysical Properties

**Figure 8:**
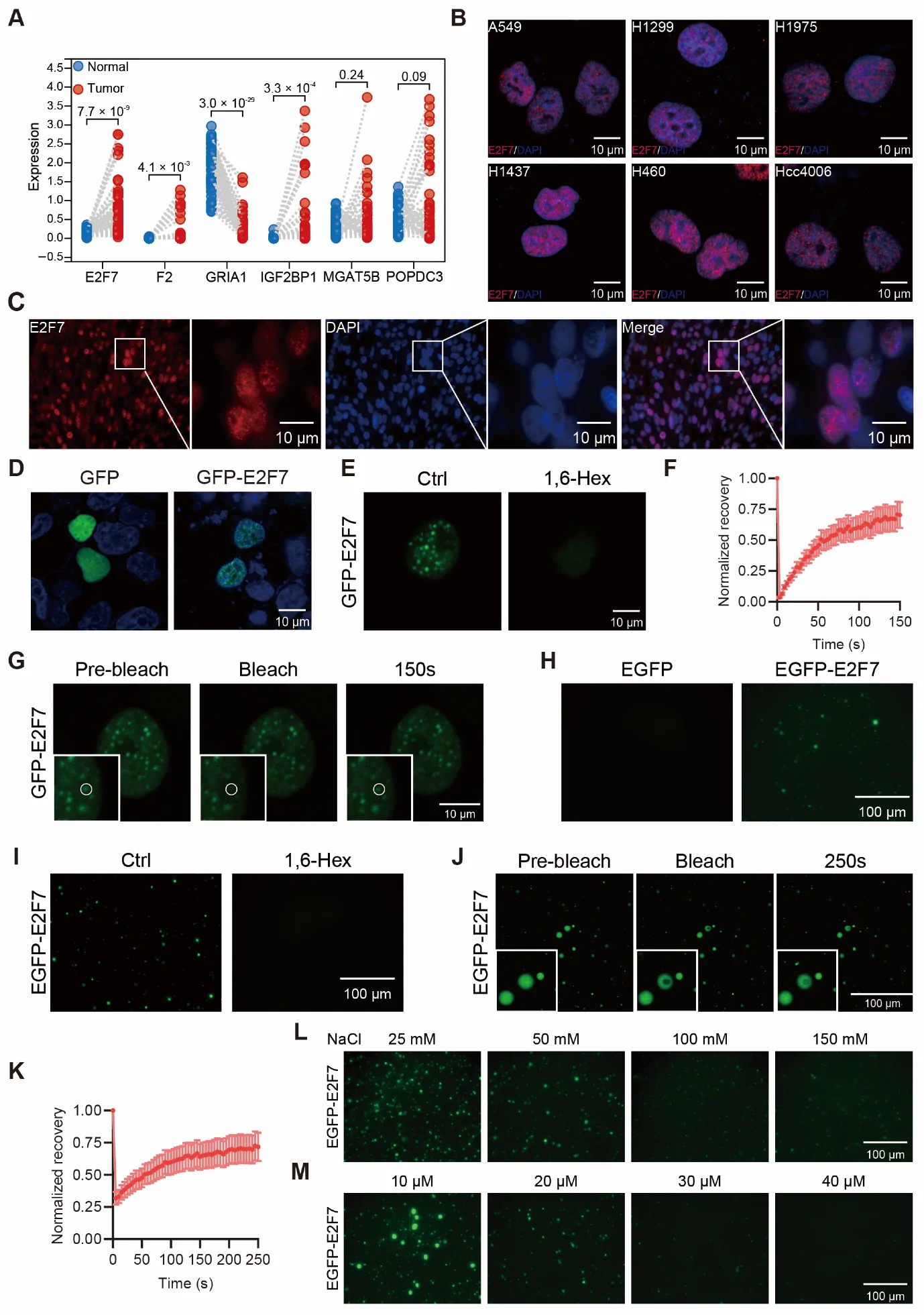
E2F7 forms dynamic condensates through liquid–liquid phase separation. (**A**) Comparison of the expression levels of the six MLORS genes between tumor and adjacent normal tissues in the TCGA-LUAD cohort. (**B**) Immunofluorescence images showing endogenous E2F7 nuclear puncta across LUAD cell lines. Scale bars, 10 μm. (**C**) Immunofluorescence images showing E2F7 localization in LUAD tissue specimens. (**D**) Confocal microscopy images showing condensate formation in A549 cells expressing GFP-E2F7 or the GFP control. Scale bars, 10 μm. (**E**) Time-lapse fluorescence imaging showing the dissolution of GFP-E2F7 puncta following treatment with 10% 1,6-hexanediol. Scale bars, 10 μm. (**F**,**G**) FRAP analysis of GFP-E2F7 condensates in A549 cells, showing fluorescence-recovery kinetics (**F**) and representative images (**G**). Scale bars, 10 μm. (**H**) Representative fluorescence images of EGFP and EGFP-E2F7 protein droplets formed in the presence of 10% PEG 8000. Scale bars, 100 μm. (**I**) Representative fluorescence images of EGFP-E2F7 protein droplets before and after treatment with 1,6-hexanediol. Scale bars, 100 μm. (**J**,**K**) FRAP analysis of EGFP-E2F7 condensates formed in vitro, showing fluorescence-recovery kinetics (**J**) and representative images (**K**). Scale bars, 100 μm. (**L**,**M**) Representative fluorescence images of EGFP-E2F7 condensates under different NaCl concentrations (**L**) and protein concentrations (**M**). Scale bars, 100 μm.

The six genes comprising the MLORS signature exhibited expression patterns broadly consistent with their corresponding risk associations in LUAD tissues. *E2F7*, *IGF2BP1*, and *F2* were significantly upregulated in tumors, whereas *GRIA1* was downregulated, in agreement with the directions of their respective MLORS coefficients (Figure 8A).

To explore the structural basis of their LLPS potential, we used AlphaFold to predict the structures of all six proteins and identified putative intrinsically disordered regions (IDRs) in each protein. E2F7 contained the most extensive predicted IDRs among the six MLORS components (Figure S5B). Analysis of Human Protein Atlas immunofluorescence images further revealed punctate intracellular localization patterns for E2F7, IGF2BP1, MGAT5B, and POPDC3, consistent with biomolecular condensate formation (Figure S5C).

Given its substantial contribution to the MLORS model, tumor-associated overexpression, and extensive predicted IDRs, E2F7 was selected for further investigation. Immunofluorescence analysis of LUAD cell lines and clinical tissue specimens showed that endogenous E2F7 formed discrete, droplet-like nuclear puncta (Figure 8B,C). Consistently, ectopic expression of GFP-E2F7 generated prominent nuclear puncta in cultured cells (Figure 8D). Treatment with 1,6-hexanediol rapidly disrupted these puncta, consistent with their dependence on weak, chemically perturbable interactions and their dynamic reversibility (Figure 8E). Fluorescence recovery after photobleaching (FRAP) analysis further revealed rapid fluorescence recovery in GFP-E2F7 puncta, consistent with dynamic exchange of E2F7 molecules between the condensates and the surrounding nucleoplasm (Figure 8F,G).

To determine whether E2F7 could undergo phase separation autonomously, we purified recombinant EGFP-E2F7 and reconstituted condensate formation in vitro. In the presence of 10% polyethylene glycol (PEG) 8000, purified EGFP-E2F7 spontaneously formed micron-scale droplets, whereas the control protein did not produce comparable structures (Figure 8H). These droplets were disrupted by 1,6-hexanediol and displayed fluorescence recovery after photobleaching, further supporting the liquid–liquid phase-separation properties of the condensates (Figure 8I–K). We next examined the effects of protein and salt concentration on droplet formation. Droplet formation increased with increasing EGFP-E2F7 concentration, whereas it decreased progressively as the NaCl concentration in the reaction buffer increased (Figure 8L,M).

Together, these cellular and biochemical analyses support the ability of E2F7 to form dynamic condensates with LLPS-consistent properties, providing experimental evidence for its potential involvement in the biological processes represented by the MLORS model.

### 2.9. IDR-Dependent Condensate Formation and Oncogenic Functions of E2F7

**Figure 9:**
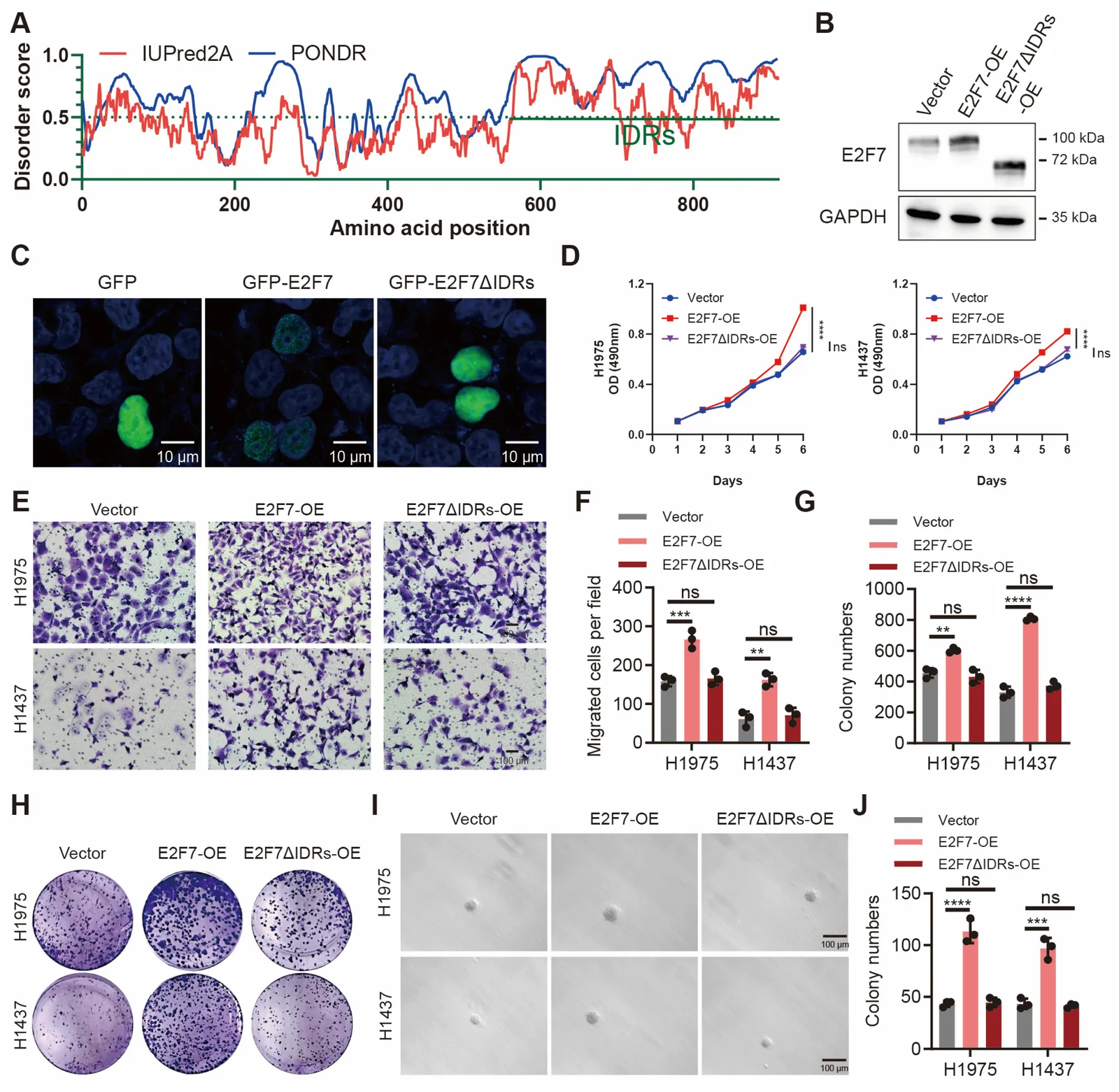
IDR-dependent condensate formation and oncogenic functions of E2F7. (**A**) Prediction of IDRs in E2F7 using the IUPred2A and PONDR algorithms, with amino acids 564–911 highlighted. (**B**) Western blot analysis confirming the expression of GFP-E2F7 and GFP-E2F7ΔIDRs. GAPDH was used as the loading control. (**C**) Confocal microscopy images showing condensate formation in cells expressing GFP-E2F7 or GFP-E2F7ΔIDRs. Scale bars, 10 μm. (**D**) MTT assay evaluating cell proliferation following overexpression of GFP-E2F7 or GFP-E2F7ΔIDRs. (**E**,**F**) Transwell assays evaluating cell migration in cells expressing GFP-E2F7 or GFP-E2F7ΔIDRs. (**G**,**H**) Colony-formation assays evaluating clonogenic growth. (**I**,**J**) Soft-agar colony-formation assays evaluating anchorage-independent growth in cells expressing the empty vector, GFP-E2F7, or GFP-E2F7ΔIDRs. ** represents *p* values < 0.01; *** represents *p* values < 0.001; and **** represents *p* values < 0.0001.

To investigate the structural basis of E2F7 condensate formation, disorder-propensity and energy-score predictions were generated using IUPred2A and PONDR. These analyses identified amino acids 564–911 as a prominent intrinsically disordered region (IDR), which was therefore selected for deletion analysis (Figure 9A). Western blotting confirmed the expression of both GFP-E2F7 and GFP-E2F7ΔIDRs (Figure 9B).

We next examined whether deletion of this predicted IDR affected E2F7 condensate formation. GFP-E2F7 formed prominent nuclear condensates, whereas deletion of the predicted IDR resulted in a more diffuse intracellular distribution (Figure 9C). We then assessed the functional consequences of this alteration. Expression of GFP-E2F7 significantly increased LUAD cell proliferation, migration, and clonogenic growth, as assessed by MTT, Transwell, and colony-formation assays, respectively (Figure 9D–H). By contrast, these effects were markedly attenuated in cells expressing GFP-E2F7ΔIDRs. In agreement with these findings, soft-agar assays showed that GFP-E2F7, but not GFP-E2F7ΔIDRs, significantly promoted anchorage-independent growth (Figure 9I,J).

Collectively, the imaging and functional data suggest that the predicted IDR contributes to E2F7 condensate formation and is associated with its oncogenic effects in LUAD cells. These findings further support E2F7 as a functionally relevant component of the MLORS model. Nevertheless, additional mechanistic studies are required to clarify the precise causal relationship between E2F7 condensate formation and the individual oncogenic phenotypes observed here.

## 3. Discussion

This study advances the understanding of liquid–liquid phase separation (LLPS)-associated biology in lung adenocarcinoma by providing a systems-level characterization of transcriptionally inferred MLO-associated activity patterns and their clinical relevance [37]. We identified coordinated MLO-associated transcriptional patterns in LUAD and demonstrated their associations with immune-related features, genomic alterations, predicted therapeutic response profiles, and patient survival. We further developed the Membraneless Organelle Risk Score (MLORS), which integrated MLO-associated gene-expression patterns and prognostic information across multiple cohorts. In addition, mechanistic experiments identified E2F7 as a representative MLORS component that formed dynamic condensates and promoted malignant phenotypes in LUAD cells. Together, these findings provide a framework linking MLO-associated transcriptional programs with LUAD heterogeneity, prognostic stratification, and potential therapeutic hypotheses.

Importantly, our study provides mechanistic support for selected components of these systems-level associations by identifying E2F7 as an LLPS-associated oncogenic regulator in LUAD. Previous studies have demonstrated that *E2F7* is aberrantly upregulated in multiple solid tumors and is associated with poor prognosis and therapeutic resistance [38,39,40]. In hepatocellular carcinoma, E2F7 promotes drug resistance through TSC1-mediated inhibition of mTORC1 while stabilizing HIF-1α to promote angiogenesis [40]. Moreover, its involvement in DNA damage repair, cisplatin resistance, and immune regulation suggests that E2F7 broadly contributes to the therapeutic response across multiple cancer types [41,42,43]. However, whether LLPS underlies these diverse biological functions has remained unknown. Here, we demonstrate for the first time that E2F7 undergoes LLPS and that IDR-dependent condensate formation is required for its tumor-promoting activity in LUAD, thereby identifying LLPS as a previously unrecognized mechanism underlying E2F7 function.

Mechanistically, our findings further demonstrate that the intrinsically disordered region (IDR) of E2F7 is indispensable for condensate formation and oncogenic activity. These observations provide a mechanistic basis for MLORS by suggesting that its prognostic performance reflects the coordinated activity of LLPS-dependent regulatory proteins rather than isolated gene expression changes. More broadly, our results support a model in which coordinated MLO-associated transcriptional programs may reflect the integrated oncogenic output of multiple phase-separating regulators. This raises the possibility that the condensome may function as a higher-order regulatory network linking genome regulation, immune remodeling, and therapeutic vulnerability in LUAD. However, the extent to which the transcriptional patterns identified in this study correspond to coordinated changes in condensome organization or function remains to be determined.

The relationship between TMB and immune-related features in our cohort also warrants careful consideration. The MLOs-H and high-MLORS groups exhibited higher TMB but lower immune- and stromal-related scores and lower expression of several immune checkpoint genes. This apparent discrepancy suggests that TMB and immune-related transcriptional features may not always be positively coupled in LUAD. One possible explanation is that MLO-associated transcriptional states and TMB may be partially decoupled with respect to immune recognition. In this model, TMB may reflect the quantity of somatic mutations, whereas MLO-associated transcriptional programs may influence antigen processing, antigen presentation, immune-cell recruitment, or other processes that determine whether tumor-derived antigens are effectively recognized. This hypothesis is consistent with the recognized limitations of TMB as a standalone biomarker for predicting immune-checkpoint inhibitor efficacy, because TMB does not fully capture neoantigen quality, clonality, antigen presentation, or the broader immune contexture of tumors [44,45]. Nevertheless, the proposed TMB–MLO decoupling remains speculative and requires validation using independent clinical cohorts, neoantigen analyses, spatial immune profiling, and functional immune-response assays.

Several limitations should be acknowledged. First, MLO-associated activity was inferred from the transcriptional enrichment of curated MLO-associated gene sets using ssGSEA. These scores capture coordinated gene-expression programs associated with MLOs but do not directly measure the physical abundance, assembly, material properties, spatial organization, or biochemical activity of membraneless organelles. Accordingly, the MLOs-H and MLOs-L groups should be interpreted as transcriptionally defined molecular patterns rather than as experimentally confirmed high- or low-abundance MLO states. Direct validation using imaging, proteomic, biophysical, or spatial approaches will be required to determine whether the inferred transcriptional patterns correspond to differences in MLO formation or function.

Second, the immune-related interpretations in this study were based primarily on computational analyses of bulk RNA-seq data. ESTIMATE, CIBERSORT, and related deconvolution approaches provide indirect estimates of immune and stromal components and cannot directly resolve individual cell populations, cellular states, spatial localization, or cell–cell interactions. Their estimates may also be affected by tumor purity, reference-signature composition, cellular heterogeneity, overlapping transcriptional programs, technical variation, and the presence of malignant cells expressing immune-related genes. Therefore, the inferred immune-exclusion or immune-suppression profiles observed in the MLOs-H and high-MLORS groups do not constitute direct evidence of immune-cell abundance, spatial exclusion, or functional suppression. These findings require validation using orthogonal approaches, such as single-cell or spatial transcriptomics, multiplex immunohistochemistry, flow cytometry, or other histological methods.

Third, drug-sensitivity analyses were based on cell-line pharmacogenomic reference data and therefore provide correlative, hypothesis-generating predictions rather than evidence of clinical treatment efficacy. The associations identified by oncoPredict, including higher predicted sensitivity to EGFR-targeted agents and the MET inhibitor savolitinib in selected groups and increased predicted susceptibility to CDK4/6 inhibition in high-risk tumors, should therefore be considered exploratory rather than clinically actionable. These therapeutic associations require validation in independent clinical cohorts and, ideally, prospective trials.

Finally, although E2F7 was selected as a representative LLPS regulator for mechanistic investigation, additional MLORS components require functional validation to determine whether LLPS represents a convergent feature across the entire signature. The model was developed and evaluated primarily in retrospective cohorts, and prospective multicenter validation will be required to determine its generalizability and clinical utility. Future integration of spatial transcriptomics, proteomics, single-cell sequencing, and functional perturbation approaches will be essential for defining how dynamic condensate states may influence immune regulation and therapeutic response.

Collectively, our findings establish transcriptionally inferred MLO-associated patterns as a previously underrecognized organizational dimension of LUAD biology that is associated with immune-related features, genomic alterations, prognosis, and predicted therapeutic response. By integrating systems-level transcriptional profiling with mechanistic validation of E2F7, this study provides a biological framework for incorporating LLPS-associated regulatory programs into future precision oncology strategies for LUAD. The proposed relationships among MLO-associated transcriptional states, TMB, immune contexture, and therapeutic response should be regarded as testable hypotheses rather than definitive causal mechanisms. Further clinical, spatial, biophysical, and functional studies will be necessary to determine how MLO-associated programs contribute to LUAD progression and whether they can be translated into clinically actionable strategies.

## 4. Materials and Methods

### 4.1. Data Acquisition and Preprocessing

RNA sequencing data (FPKM) and corresponding clinical annotations for the TCGA-LUAD cohort were obtained from The Cancer Genome Atlas. Independent validation cohorts (GSE30219, GSE31210, GSE50081) were retrieved from the Gene Expression Omnibus [46,47,48]. Somatic mutation data (MAF format) were obtained from TCGA and analyzed for tumor mutational burden (TMB) and driver gene alterations. Drug response data were derived from the GDSC and CTRP databases for predictive modeling [49,50].

### 4.2. MLO Gene Set Curation and Activity Quantification

Experimentally validated LLPS-driven membraneless organelles (MLOs) and their core component genes were curated from PhaSepDB [26], yielding 24 MLO-associated gene sets. Single-sample gene set enrichment analysis (ssGSEA) was performed using the GSVA package to quantify the transcriptional enrichment of MLO-associated gene sets at the individual-sample level. These ssGSEA scores represent transcriptome-based molecular activity signatures and should not be interpreted as direct measurements of the physical abundance, assembly state, material properties, or biochemical activity of membraneless organelles. Accordingly, the term “MLO activity” in this study refers to transcriptionally inferred MLO-associated activity rather than directly measured organelle abundance or biophysical activity.

### 4.3. Molecular Subtyping and Functional Annotation

Unsupervised consensus clustering based on transcriptionally inferred, prognosis-associated MLO activity scores was performed with the ConsensusClusterPlus R package (k-means partitioning; Euclidean distance; 500 resampling iterations, with 80% of samples and all features drawn per iteration; maximum evaluated cluster number, k = 9; random seed 123456) to identify molecular subtypes characterized by coordinated MLO-associated transcriptional patterns. The optimal number of clusters was determined from the consensus CDF, the delta-area plot, and the consensus matrix heatmap, and a two-cluster solution (k = 2) was selected for downstream analysis. Differential expression analysis between subtypes was performed using limma. Functional enrichment analyses, including Gene Ontology (GO) and Kyoto Encyclopedia of Genes and Genomes (KEGG) pathways, were conducted using clusterProfiler, with a false discovery rate (FDR) < 0.05 considered significant.

### 4.4. Immune Microenvironment and Immunogenomic Analysis

Tumor immune and stromal components were computationally estimated from bulk RNA-seq expression profiles using the ESTIMATE algorithm [51]. Relative immune-cell abundance was inferred by computational deconvolution using multiple reference-based methods, including xCell, TIMER, quanTIseq, MCP-counter, EPIC, CIBERSORT, and CIBERSORT-ABS [52]. Immune pathway activity was quantified using ssGSEA. Associations with tumor mutational burden (TMB) and microsatellite instability (MSI) were evaluated [44,53]. Because these approaches infer cellular composition from bulk transcriptomic mixtures, the resulting scores represent computational estimates rather than direct measurements of immune-cell abundance or spatial organization.

### 4.5. Prognostic Modeling and Risk Score Construction

Genes differentially expressed between the MLOs-high and MLOs-low subtypes were screened using the criteria (|log_2_FC| > 1) and FDR < 0.01. Univariate Cox regression analysis was used to identify prognosis-associated genes. The resulting 117 genes were intersected with the genes available in the GEO validation cohorts, yielding 75 genes that were used as input for the machine-learning analysis. An integrative machine-learning analysis was performed using the Mime1 R package, which implements ten survival algorithms, combining these algorithms with different feature-selection strategies and hyperparameters yielded 101 candidate models, all evaluated under 10-fold cross-validation [54]. Among the candidate models, all of which achieved concordance index (C-index) values above 0.5 in the TCGA training cohort, the Random Survival Forest (RSF) model was selected as the optimal prognostic model because it combined a high C-index (0.670) with the fewest genes (six genes). RSF directly generated the final six-gene signature used to calculate the Membraneless Organelle Risk Score (MLORS) [55]. The optimal MLORS cutoff was determined in the TCGA-LUAD training cohort by maximizing the difference in overall survival between the resulting risk groups, and was applied unchanged to all validation cohorts (GSE30219, GSE31210, GSE50081) to minimize potential data leakage. Patients were then classified into high- and low-risk groups according to this cutoff. Model performance was evaluated using Kaplan–Meier survival and time-dependent receiver operating characteristic curves.

To assess the robustness of the RSF model and to address the requirement that feature selection be performed within each training fold, a nested cross-validation (NCV) framework was implemented as a supplementary analysis. The NCV used the same 75-gene set as the 101-model comparison. RSF hyperparameters (ntree = 1000, nodesize = 5, splitrule = “logrank”) were kept unchanged. The NCV comprised an outer 5-fold cross-validation repeated five times (25 outer folds) and an inner 10-fold cross-validation for model selection within each training fold. In each outer fold, the inner 10-fold CV was used to select the optimal model, which was then refitted on the full training fold and evaluated on the held-out test fold. The outer-test C-index was 0.628 ± 0.067 (range 0.513–0.735; per-repeat mean 0.617–0.637), 0.042 lower than the single-layer 10-fold CV C-index (0.670). This decrease is consistent with the correction for optimistic bias by NCV, and the outer-test C-index remained well above 0.5, indicating robust generalizability of the RSF pipeline. The 117-gene univariate Cox screening was performed on the full TCGA training cohort and was not re-executed within each training fold; this NCV therefore evaluates the generalizability of model comparison and RSF fitting given a fixed 75-gene set, rather than that of the complete feature-selection pipeline.

### 4.6. Genomic Alteration and Drug Sensitivity Analysis

Somatic mutation profiles were analyzed using maftools, and subtype-specific mutation frequencies were compared using Fisher’s exact test. Cell-line-based drug sensitivity was predicted using the oncoPredict framework based on the GDSC and CTRP reference datasets. These predictions represent correlative associations between transcriptomic profiles and pharmacological response in reference cell-line models and were not intended to provide direct estimates of clinical treatment efficacy.

### 4.7. Cell Culture and Plasmid Construction

Human lung cancer cell lines A549, H1299, H1975, H1437, H460, and HCC4006 were obtained from the American Type Culture Collection. Cells were cultured in DMEM supplemented with 10% fetal bovine serum at 37 °C in a humidified atmosphere containing 5% CO_2_.

The full-length *E2F7* sequence and an *E2F7* mutant lacking the predicted intrinsically disordered region (amino acids 564–911) were cloned into GFP-tagged pCDH vectors and then designated GFP-E2F7 and GFP-E2F7ΔIDRs, respectively. An empty GFP-tagged pCDH vector was used as the control. All constructs were verified using DNA sequencing. Transient transfections were performed using Lipofectamine 3000 according to the manufacturer’s instructions.

### 4.8. Functional Assays

Cell proliferation was assessed using MTT and colony formation assays. Cell migration was evaluated using Transwell assays, and anchorage-independent growth was examined using soft agar colony formation assays. Cells transfected with the empty vector, GFP-E2F7, or GFP-E2F7ΔIDRs were compared under the same experimental conditions. All functional experiments were performed in biological triplicate, and representative results are shown.

### 4.9. Western Blotting

Cells were lysed in RIPA buffer containing protease inhibitors. Equal amounts of protein were separated by SDS–PAGE and transferred to PVDF membranes. After blocking with 5% non-fat milk, the membranes were incubated overnight at 4 °C with antibodies against E2F7 (Proteintech, Cat. No. 24489-1-AP; 1:500) and GAPDH (Proteintech, Cat. No. 60004-1-Ig; 1:5000), followed by incubation with HRP-conjugated secondary antibodies. Protein bands were detected using enhanced chemiluminescence. GAPDH served as the loading control. The E2F7 antibody recognized both the GFP-E2F7 and GFP-E2F7ΔIDRs constructs. All experiments were performed using three independent biological replicates.

### 4.10. Recombinant Protein Expression and Purification

For in vitro phase-separation assays, the codon-optimized EGFP-E2F7 sequence was cloned into the His-tagged pET-28a(+) vector and expressed in *Escherichia coli* BL21(DE3) cells. Protein expression was induced with 0.5 mM IPTG at 16 °C for 16 h when the culture reached an OD_600_ of 0.6–1.0. Cells were lysed by sonication, and His-tagged EGFP-E2F7 was purified using a Ni-NTA His-tagged Protein Purification Kit (Kangwei, Cat. No. CW0894S; Beijing, China). Protein concentration was determined using the BCA assay. The purified protein was concentrated, desalted, and stored at 4 °C until use.

### 4.11. Phase Separation Assays

Intrinsically disordered regions were predicted using IUPred2A and PONDR [56,57], and structural models were obtained from AlphaFold [58].

Cells expressing GFP-E2F7, GFP-E2F7ΔIDRs, or the empty vector were examined by confocal microscopy to assess subcellular localization and nuclear condensate formation. GFP fluorescence was directly used to visualize the tagged constructs.

Purified EGFP-E2F7 protein was diluted in phase separation buffer containing 10% PEG 8000 and NaCl at final concentrations of 25, 50, 100, or 150 mM. The buffer was adjusted to pH 7.4. A 10 μL aliquot of each reaction mixture was placed on a coverslip and imaged by confocal microscopy (Zeiss LSM780; Carl Zeiss, Jena, Germany).

FRAP was performed in living cells and in vitro condensates. Defined regions within GFP-positive nuclear condensates or in vitro EGFP-E2F7 condensates were photobleached to approximately 25% of the initial fluorescence, and images were acquired every 5 s for 5 min. Fluorescence recovery was corrected for background fluorescence and acquisition-related photobleaching using an unbleached reference region, normalized to the prebleaching intensity, and analyzed using GraphPad Prism 10.5.0.

For dissolution assays, 1,6-hexanediol was added to a final concentration of 10%, and condensate dissolution was monitored under identical imaging conditions.

### 4.12. Immunofluorescence and Confocal Imaging

Public immunofluorescence images were obtained from the Human Protein Atlas. Cells and tissue sections were fixed, permeabilized, and blocked before incubation overnight at 4 °C with an anti-E2F7 antibody (Proteintech, Cat. No. 24489-1-AP; 1:500; Wuhan, China), followed by incubation with a fluorophore-conjugated secondary antibody. Nuclei were counterstained with DAPI, and images were acquired by confocal microscopy under identical settings.

A commercial human lung adenocarcinoma tissue microarray (ZL-LUC601; Shanghai Weiao Biotechnology Co., Ltd., Shanghai, China) was used for tissue immunofluorescence analysis. Its use was approved by the Ethics Committee of Shanghai Weiao Biotechnology Co., Ltd. (Approval Code: IRBS-LUC601-25-048; Approval Date: 20 February 2025). All specimens were de-identified, and the study was conducted in accordance with the principles of the Declaration of Helsinki.

### 4.13. Statistical Analysis

All statistical analyses were performed using R version 4.5.1. Differences between two groups were evaluated using the Wilcoxon rank-sum test or Fisher’s exact test, as appropriate. Survival differences were assessed using Kaplan–Meier analysis and the log-rank test. Correlations were evaluated using Spearman’s correlation. All tests were two-sided, and *p* < 0.05 was considered statistically significant. * represents *p* values < 0.05; ** represents *p* values < 0.01; *** represents *p* values < 0.001; and **** represents *p* values < 0.0001.

## 5. Conclusions

This study characterized transcriptionally inferred MLO-associated activity signatures in lung adenocarcinoma and identified two groups with distinct immune, genomic, and survival profiles. We further developed the Membraneless Organelle Risk Score (MLORS), which consistently stratified overall survival across multiple retrospective cohorts and was associated with tumor immune characteristics, mutational burden, and predicted drug sensitivity. These findings support transcriptionally inferred MLO-associated patterns as a potential dimension of LUAD heterogeneity and suggest that MLORS may provide prognostic information complementary to tumor mutational burden and conventional clinicopathological variables.

Among the MLORS components, E2F7 formed IDR-dependent phase-separation condensate and promoted LUAD cell proliferation, migration, and anchorage-independent growth in vitro. Although these observations are consistent with LLPS behavior, additional biophysical validation is required to establish bona fide phase separation and define its underlying molecular mechanisms. Prospective clinical validation and further functional investigation of the remaining MLORS components will be necessary before clinical application.

## Data Availability

The TCGA-LUAD data analyzed in this study are available from the Genomic Data Commons (https://portal.gdc.cancer.gov/). The GSE30219, GSE31210, and GSE50081 datasets are available from the Gene Expression Omnibus (https://www.ncbi.nlm.nih.gov/geo/, accessed on 25 July 2025). The data that support the findings of this study are available from the corresponding author upon reasonable request.

## Supplementary Materials

The following supporting information can be downloaded at https://www.mdpi.com/article/10.3390/ijms272110008/s1.

## Abbreviations

ALK	Anaplastic lymphoma kinase
AUC	Area under the curve
C-index	Concordance index
CTRP	Cancer Therapeutics Response Portal
DMEM	Dulbecco’s modified Eagle’s medium
EGFR	Epidermal growth factor receptor
FDR	False discovery rate
FRAP	Fluorescence recovery after photobleaching
GDSC	Genomics of Drug Sensitivity in Cancer
GFP	Green fluorescent protein
GO	Gene Ontology
IDR	Intrinsically disordered region
KEGG	Kyoto Encyclopedia of Genes and Genomes
LLPS	Liquid–liquid phase separation
LUAD	Lung adenocarcinoma
MAF	Mutation Annotation Format
MHC	Major histocompatibility complex
MLO	Membraneless organelle
MLORS	Membraneless Organelle Risk Score
MLOs-H	MLOs-high subtype
MLOs-L	MLOs-low subtype
MSI	Microsatellite instability
NCV	Nested cross-validation
OS	Overall survival
PONDR	Predictor of Naturally Disordered Regions
ROC	Receiver operating characteristic
RSF	Random Survival Forest
ssGSEA	Single-sample gene set enrichment analysis
TCGA	The Cancer Genome Atlas
TMB	Tumor mutational burden
TNM	Tumor–node–metastasis
