# Supplementary material for: LLPS-Driven Membraneless Organelle Activity Landscape Reveals Molecular Subtypes, Immune States, and Prognostic Stratification in Lung Adenocarcinoma
Source: Int J Mol Sci. 2026 Nov 8;27(21):10008. doi: 10.3390/ijms272110008 (PMC13764032; doi:10.3390/ijms272110008)
Supplement: Supplementary file 1 [file ijms-27-10008-s001.zip › Supplementary_Figures.pdf]

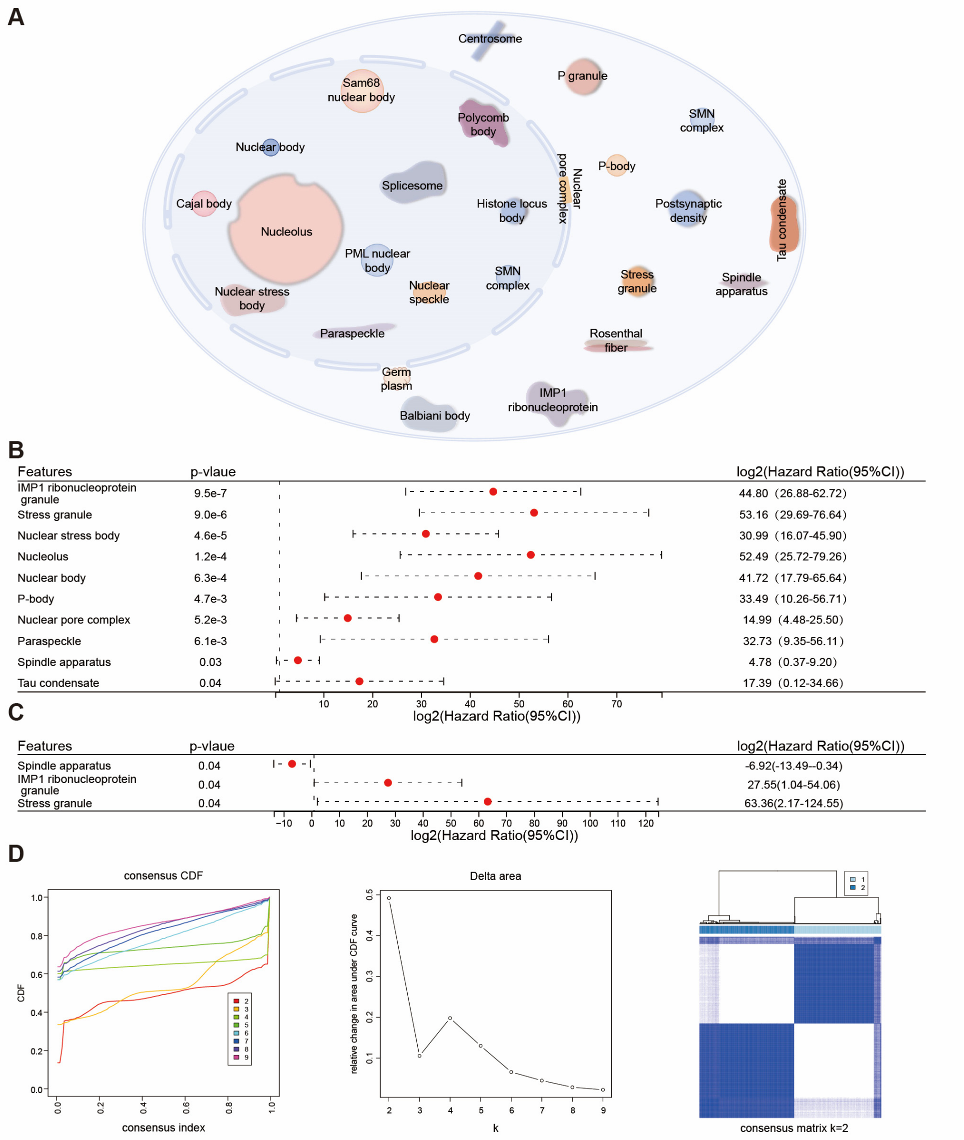

Figure S1. Prognostic relevance of MLO-associated activity and molecular subtype identification in LUAD. (A) Schematic representation of 24 experimentally validated LLPS-associated MLOs. (B) Univariate Cox regression analysis of the association between MLO-associated activity and overall survival. (C) Multivariable Cox regression analysis of independently prognostic MLO-associated activities. (D) Unsupervised clustering of the 12 prognostic MLO ssGSEA profiles in TCGA-LUAD.

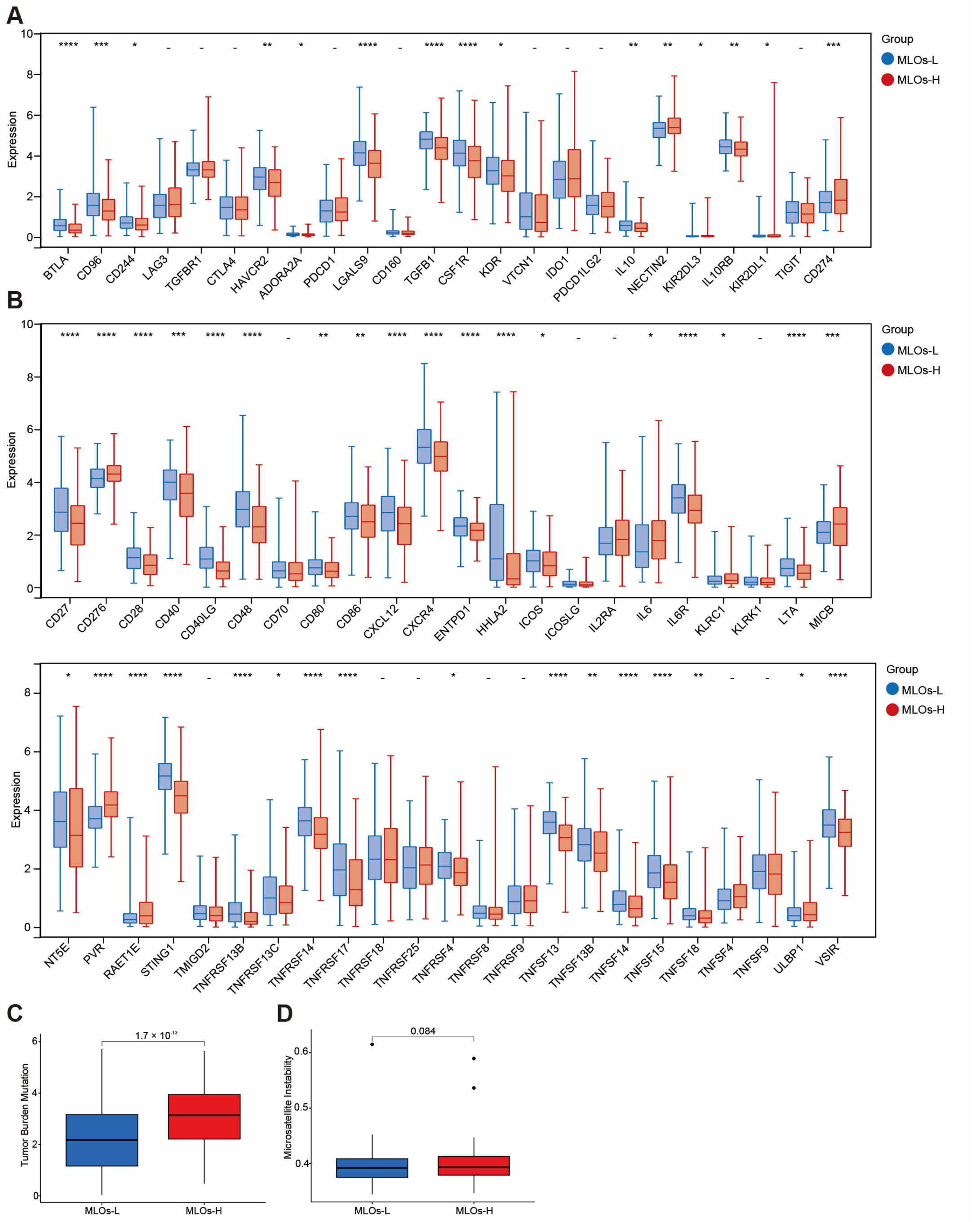

Figure S2. Immune checkpoint expression and immunogenomic features of MLO-associated LUAD subtypes. (A) Differential expression of inhibitory immune checkpoint genes between MLOs-H and MLOs-L tumors. (B) Differential expression of co-stimulatory immune checkpoint genes between the two subtypes. (C) Comparison of tumor mutational burden (TMB) between the two subtypes. (D) Comparison of microsatellite instability (MSI) status between the two subtypes. * represents p values < 0.05; ** represents p values < 0.01; *** represents p values < 0.001; and **** represents p values < 0.0001.

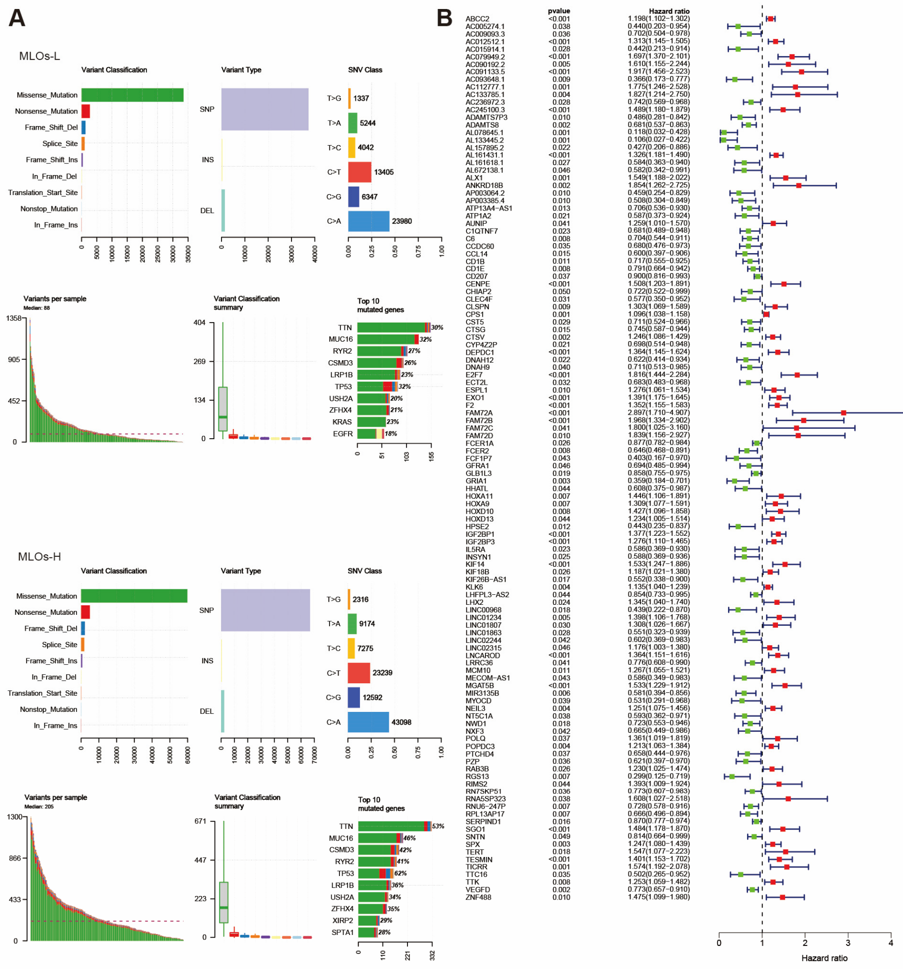

Figure S3. Genomic alterations and prognostic gene identification in MLO-associated subtypes. (A) Somatic mutation landscapes and the most frequently mutated genes in MLOs-H and MLOs-L tumors. (B) Identification of prognosis-associated genes using univariate Cox regression analysis.

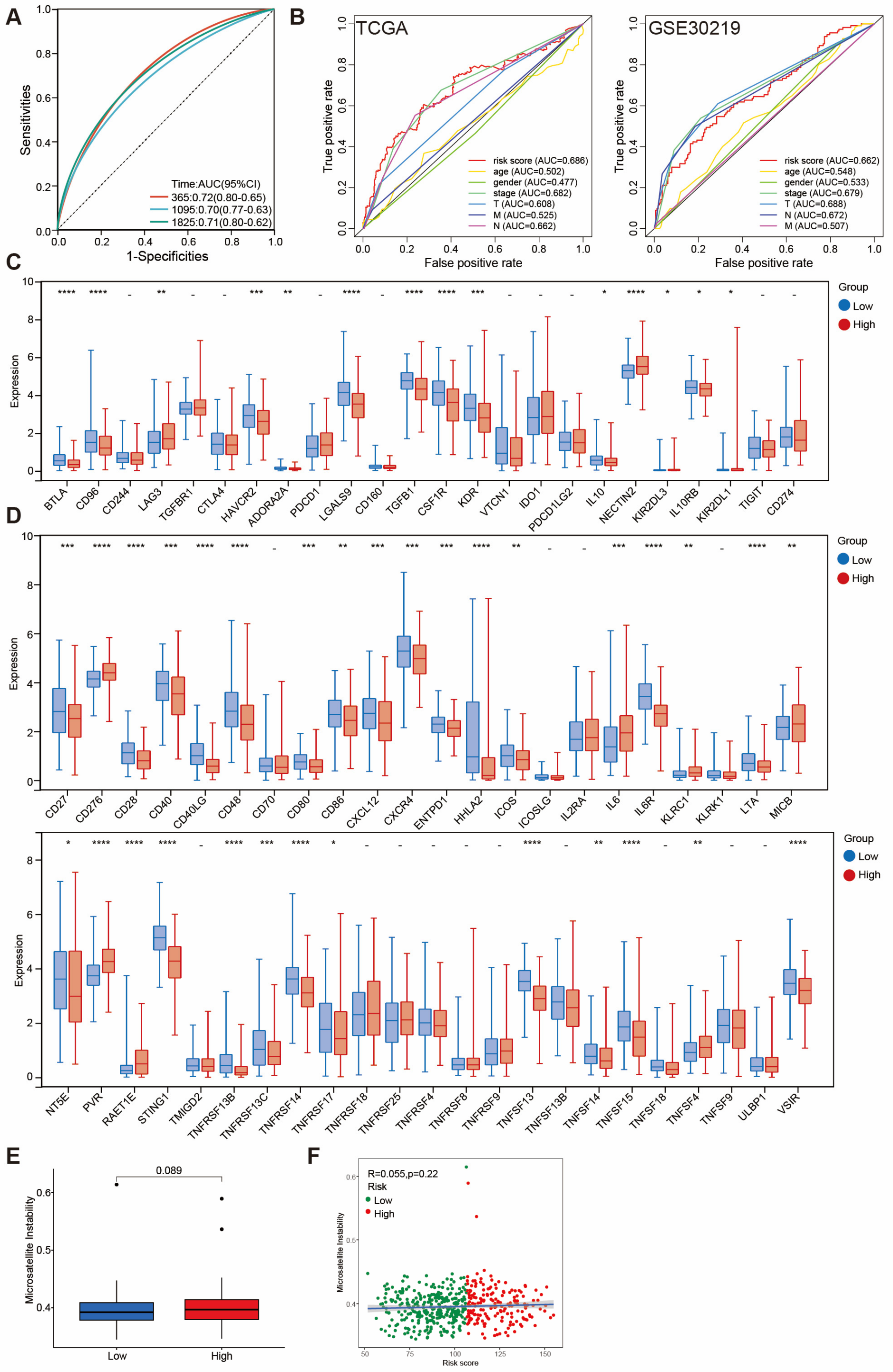

Figure S4. Prognostic performance and immunogenomic characteristics associated with MLORS. (A) Time-dependent receiver operating characteristic (ROC) curves evaluating the ability of MLORS to predict 1-, 3-, and 5-year overall survival in the training cohort. (B) Comparison of the 3-year area under the ROC curve (AUC) between MLORS and conventional clinicopathological variables. (C) Differential expression of inhibitory immune checkpoint genes between MLORS high- and low-risk groups. (D) Differential expression of co-stimulatory immune checkpoint genes between the two risk groups. (E) Comparison of microsatellite instability (MSI) status between the two risk groups. (F) Correlation between MLORS and MSI status. * represents p values < 0.05; ** represents p values < 0.01; *** represents

p values < 0.001; and **** represents p values < 0.0001.

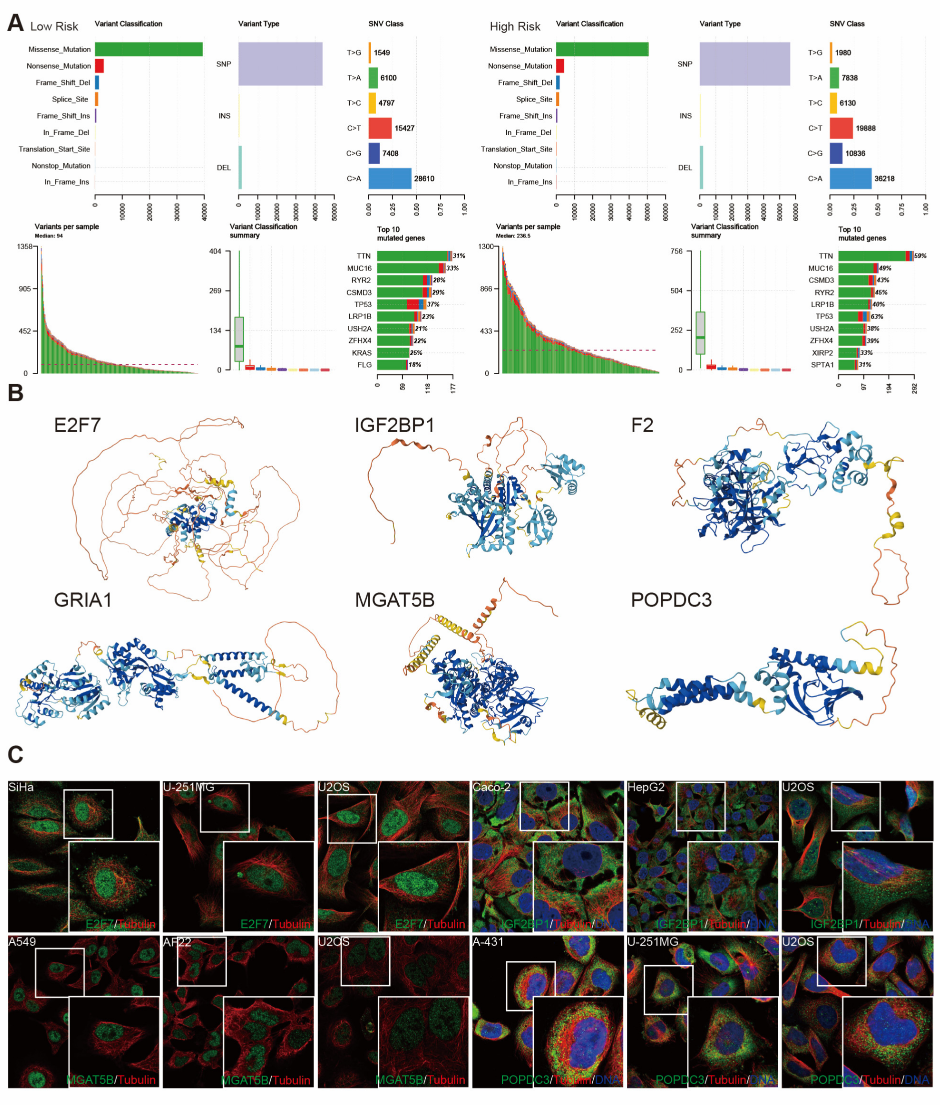

Figure S5. Predicted structural and subcellular localization features of MLORS components. (A) Somatic mutation landscapes and the most frequently altered genes in the MLORS high- and low-risk groups. (B) AlphaFold-predicted protein structures of the six MLORS components. (C) Representative immunofluorescence images of E2F7, IGF2BP1, MGAT5B, and POPDC3 obtained from the Human Protein Atlas.
